# Using MUSIC and CC(CO)NH for Backbone Assignment of Two Medium-Sized Proteins Not Fully Accessible to Standard 3D NMR

**DOI:** 10.3390/molecules19078890

**Published:** 2014-06-26

**Authors:** Annette K. Brenner, Nils Åge Frøystein

**Affiliations:** 1 Department of Chemistry, University of Bergen, PO Box 7800, 5020 Bergen, Norway;E-Mail: nils.froystein@kj.uib.no; 2 Department of Clinical Science, University of Bergen, PO Box 7804, 5020 Bergen, Norway

**Keywords:** protein NMR spectroscopy, backbone assignment, CC(CO)NH, MUSIC

## Abstract

The backbone assignment of medium-sized proteins is rarely as straightforward as that of small proteins, and thus often requires creative solutions. Here, we describe the application of a combination of standard 3D heteronuclear methods with CC(CO)NH and a variety of MUltiplicity Selective In-phase Coherence transfer (MUSIC) experiments. Both CC(CO)NH and MUSIC are, in theory, very powerful methods for the backbone assignment of proteins. Due to low sensitivity, their use has usually been linked to small proteins only. However, we found that combining CC(CO)NH and MUSIC experiments simplified the assignment of two challenging medium-sized proteins of 13 and 19.5 kDa, respectively. These methods are to some extent complementary to each other: CC(CO)NH acquired with a long isotropic mixing time can identify amino acids with large aliphatic side chains. Whereas the most sensitive MUSIC experiments identify amino acid types that cannot be detected by CC(CO)NH, comprising the residues with acid and amide groups, and aromatic rings in their side chains. Together these methods provide a means of identifying the majority of peaks in the 2D ^15^N HSQC spectrum which simplifies the backbone assignment work even for proteins, e.g., small kinases, whose standard spectra resulted in little spectral resolution and low signal intensities.

## 1. Introduction

The backbone assignment of small, isotope-labelled proteins (<*ca*. 10 kDa) is usually quite easily accomplished by the use of 3D heteronuclear standard methods. Important factors that complicate the resonance assignment for medium-sized (*ca*. 10–25 kDa) proteins, which include small kinases or domains of larger kinases, are signal degeneracy, missing NH-peaks due to increased line-widths and reduced signal to noise ratios due to insufficient suppression of the dominant water signal [1]. Thus, the standard experiments are often no longer sufficient.

There are three main strategies that may allow for the backbone assignment of medium-sized proteins with reasonable H^N^ chemical shift distribution: the use of high-field NMR spectroscopy, partial sample deuteration and the use of amino acid type specific methods that can be divided into five sub-categories. (i) The straightforward approach is to use the C*^α^* and C^β^ chemical shift values which can lead to the certain identification of glycine, alanine and the serine/threonine pair. The other amino acids cannot be identified specifically, but probability scores can be obtained to narrow down the possibilities, especially if one was able to link several spin systems together [2] and if the secondary structures of the protein are known [3]. Additionally, assignment programs like Cara make use of the chemical shift statistics which are updated regularly by the biomagnetic resonance bank [4] (ii) The selective labelling [5,6] or unlabelling [7] of specific amino acids types result in ^15^N HSQC sub-spectra which either only contain the labelled or which lack the selectively unlabelled residues. The former approach requires the use of expensive ^13^C/^15^N labelled amino acids and does not facilitate the linkage of neighbouring residues [7], whereas both methods necessitate the expression and purification of several protein NMR samples; (iii) The HADAMAC [8,9] or iHADAMAC [10] experiments utilize that amino acids can be divided into seven topology classes according to the number of protons attached to their *α* and β carbons and the number and type of carbons at the *γ*-position [8]. The methods are sensitive, but two of the classes contain as many as 13 amino acids, whose further discrimination might be difficult; (iv) All aliphatic carbon chemical shifts can be determined from the TOCSY-based 3D CC(CO)NH experiment [11,12], which, at best, can lead to the unambiguous assignment of glycine, alanine, serine, threonine, valine, leucine, isoleucine, arginine, lysine and proline. Unfortunately, the CC(CO)NH method has a low inherent sensitivity, which additionally worsens with increasing molecular mass; (v) Finally, there are the 2D MUSIC experiments which selectively detect only one to three amino acid types at a time [13,14,15,16,17]. With the exception of the novel pulse-sequences designed for proline [14], all MUSIC techniques are modifications of the 3D CBCA(CO)NH and CBCANH standard methods. These 2D experiments are time-consuming if all of them are acquired and those with many transfer steps exhibit low sensitivity [10]. We want to stress that the intention of the approach in this communication is the assignment of the peaks in the ^15^N HSQC spectrum for further elucidation of e.g., ligand interaction, relaxation and temperature studies. For proteins with poor H^N^ shift distribution like intrinsically disordered proteins, or proteins with a high degree of exchange broadened peaks, alternative methods will have to be used. Strategies for these proteins include ^13^C direct detection methods, preferable combined with non-uniformly sampling to allow the acquisition of multiple (4D and 5D) dimensional spectra in a reasonable amount of time [18]. There one can, for instance, combine the 3D CCCON experiment, which links the aliphatic carbons to their C' and N^H^ chemical shifts [19], with several amino acid specific modified 3D (H)CACON spectra [20] resulting in a similar approach as that we propose, but which eventually leads to the assignment of the 2D CON spectrum instead of the ^15^N HSQC.

In this study, the backbone assignment of two medium-sized proteins, chicken brain *α*-spectrin repeat 17 (R17) [21] and human N*^α^*-acetyltransferase 50 protein (hNaa50p) [22], was mainly obtained from the combination of CC(CO)NH with various MUSIC experiments. The assignment work was challenging for different reasons: R17 (13 kDa) is all-helical which, in spite of a small degree of peak-broadening and good sensitivity of the basic 3D methods, led to high chemical shift degeneracy in the ^15^N HSQC spectrum. hNaa50p (19.5 kDa) on the other hand, showed a high degree of peak overlap both due to the number of signals and peak-broadening. Hence, the standard 3D methods were only sufficient for the sequential assignment of segments with well-resolved peaks in the ^15^N HSQC spectrum due to peak overlap that resulted in assignment ambiguities also in the third spectral dimension. However, the unassigned chemical shifts obtained from CBCA(CO)NH provided a useful starting point for an assignment strategy based on extended aliphatic carbon side chains since they allowed the use of longer CC(CO)NH mixing times suitable for the more remote carbon atoms. The combination of CC(CO)NH with MUSIC experiments thus resulted in the almost complete backbone assignment of both proteins at a moderate field strength (600 MHz) without the use of more than one protein sample. The peak intensities of the aliphatic carbons obtained from CC(CO)NH using several mixing times, and the sensitivity and specificity of a selection of 30 MUSIC experiments had been tested on the small (8.6 kDa), well-described human ubiquitin in advance. The approach of using CC(CO)NH and MUSIC in addition to standard methods might be suitable for other all-helical proteins up to *ca.* 15 kDa as well as well-structured proteins up to approximately 20–25 kDa (e.g., small kinases [23], receptor ligands [24] and intracellular mediators initiating or facilitating signalling events upstream to the kinases [25]) in cases where high-field instruments are not available and where one wants to use as few samples as possible.

## 2. Results and Discussion

### 2.1. CC(CO)NH Spectra and the Choice of Mixing Times

It was possible to identify the amino acid types from their aliphatic carbon shifts from all three spectra (mixing times of 22, 27 and 30 ms) in ubiquitin. Even though the average signal strengths appeared to be optimal with the shortest mixing time, longer mixing times have the advantage that they yield higher peak intensities for the remote carbons in the side chain, which often are most valuable for the assignment of the amino acid type. The protein concentrations of R17 and hNaa50p were only approximately 20% of that of ubiquitin and CC(CO)NH gets less sensitive with increasing molecular mass. However, it was sufficient to collect only one CC(CO)NH spectrum with the averaged long mixing time of 28 ms for these proteins, because most of the C*^α^* and the C^β^ shifts were already obtained from standard experiments in advance.

**Figure 1:**
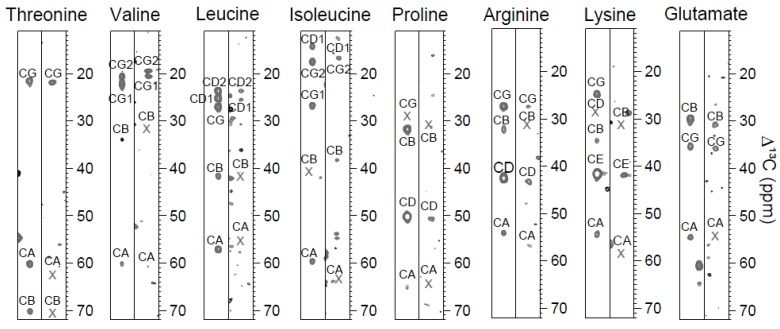
Amino acid type identification by the 3D CC(CO)NH experiment. The strips on the left and right side show spin systems of human ubiquitin and of hNaa50p, respectively. Even though CC(CO)NH is much less sensitive for larger proteins, a long mixing time allows for the observation of the remote carbons which are valuable for the assignment work. Crosses indicate chemical shifts that were determined from CBCA(CO)NH. Glutamate is shown as an example of the group that also comprises glutamine and methionine, which all have a similar chemical shift distribution.

Ten amino acid types can be directly identified by CC(CO)NH, whereas the remaining ten can be divided into three groups (Table 1). In ubiquitin, it was possible to assign almost all aliphatic carbons for all amino acid types. In R17 and especially hNaa50p, the information from CBCA(CO)NH combined with the results from CC(CO)NH could be used to identify or predict the most likely amino acid type (Figure 1). CBCA(CO)NH could determine glycine, alanine and the pair of serine and threonine, whereas CC(CO)NH was used to distinguish between the latter two amino acids. Amino acids with long aliphatic side chains were identified from the range of the C*^γ^* (V), C*^δ^* (L, P), C*^γ^* and C*^δ^* (I, R) and C^ε^ (K) chemical shifts.

**Table 1:** Amino acid type specificity of 3D CC(CO)NH using long isotropic mixing times of 27 (ubiquitin) or 28 ms. The table shows how many of the total number of an amino acid type could be identified in one small and two medium-sized proteins.

Amino Acid	Ubiquitin	Detectable Signals ^a^	
		R17	hNaa50p
G	5/5	6/6	10/10
A	2/2	11/12	7/7
S	3/3	3/4	6/6
T	7/7	5/5	4/4
I	5/5	3/3	9/12
V	4/4	6/8	8/8
L	9/9	7/9	8/13
R	4/4	4/4	5/6
K	7/7	13/13	10/12
P	2/2	-	2/4
E/Q/M	12/12	17/21	6/14
N/D/F/Y/C_ox_	9/9	11/20	11/29
H/W/C_red_	1/1	0/4	1/6

^a^ Amino acids that are predecessors of proline or of amino acids that have solvent or conformational exchanged NH-peaks were not included in the total number of that specific amino acid.

Furthermore, the group of glutamine, glutamate and methionine was determined from the positioning of their C^γ^ shift. Many of the remaining seven amino acid types lacked signals apart from C*^α^*, and could thus not be identified from CC(CO)NH. Table 1 shows how many amino acids could be detected out of the total amount of a specific type in the proteins.

### 2.2. MUSIC Spectra of Ubiquitin

Thirty different MUSIC experiments were tested on ubiquitin, and 27 of them yielded satisfactory results (Table 2). Three out of four proline-detecting experiments showed insufficient signal strengths and were not investigated further.

Other MUSIC techniques appeared to be rather insensitive, like A_ii+1, N_ii+1, and experiments (both versions) that detect threonine, leucine, arginine and lysine. Good signal strengths, on the other hand, were obtained from experiments detecting asparagine, aspartate, glutamine, glutamate, valine, isoleucine and the aromatic residues. Because most of these amino acid types are difficult to determine from CC(CO)NH, the corresponding experiments were the most promising for the assignment of the medium-sized proteins. Since the spectra from several variants can be superpositioned with each other, e.g., N_i+1, DNG_i+1, EQG_i+1 and NQ_i+1, it is possible to specifically assign most of the amino acids with acid and amide groups in their side chains.

Some MUSIC spectra contained artefacts which are in agreement with the previous reported ones, and that one should be aware of when running these experiments. The G_i+1 experiment, which is selective for CH_2_-groups directly bound to an amide, leads to weak side chain NHD-peaks of asparagine and glutamine [13]. In the G_ii+1 spectrum on the other hand, which is selective for methylene groups bound to an amine, arginine side chain signals are observable [13]. However, these peaks are usually easily identified because of their spectral positions (asparagine and glutamine) and because of aliasing into the spectrum with sign inversion (arginine). Most glycine peaks in the DNG and EQG experiments are negative, and thus easily recognized. Some of the asparagine signals tend to be negative as well. Due to varying values of the ^1^*J*_CC_ coupling constant, asparagine and aspartate cannot always be completely suppressed and might appear as weak negative artefacts in the EQG-spectra [15], but these can be identified from the superposition of the DNG- and EQG-spectra and the C^β^ chemical shift values. For the same reason, methyl signals belonging to valine, isoleucine or threonine might break through in the LA-spectra [15].

In the VIA experiments, valine tends to show a higher signal intensity than isoleucine because two *γ*-methyl groups contribute to the valine peaks [15]. In addition, these two amino acids can be easily distinguished from each other due to their differing C^β^ chemical shift values. Interestingly, in the FYH spectra the phenylalanines showed sign inversion compared to histidine and tyrosine. Since histidine has another C^β^ chemical shift range than the other two aromatics, this allowed for the direct identification of the three amino acid types in human ubiquitin.

Interestingly, even though both E24 and G53 are not observable in the ^15^N HSQC spectrum due to exchange-broadening, the two successors of these residues appear in the EQG_i+1 spectrum.

**Table 2:** Overview of 30 MUSIC experiments that were acquired on human ubiquitin.

Variant ^a^	Acquisition Time (h)	Signals	Artefacts	Comments
G_i+1 ^b^	3	5/5	N ^c^/Q ^c^	weak artefact signals
G_ii+1 ^d^	3	5/5	R ^c^	artefacts are negative if aliased
A_i+1	3	2/2		
A_ii+1	1.5	2/2		low sensitivity
S_i+1	4.5	3/3		
S_ii+1	4.5	3/3		
N_i+1	2	2/2		
N_ii+1	8	2/2		low sensitivity
NQ_i+1	2	8/8		
NQ_ii+1	2	8/8		One Q is negative
DNG_i+1	2	11/11		G and one N are negative
DNG_ii+1	2	12/12		G and one N are negative, N have lowest signal strengths
EQG_i+1	2	18/18	N	G and one Q are negative
EQG_ii+1	2	15/16	N	G and one Q are negative
TA_i+1	1.5	8/9		low sensitivity
TA_ii+1	1.5	9/9		low sensitivity
VIA_i+1	1.5	13/13		V has higher intensity than I
VIA_ii+1	1.5	13/13		V has higher intensity than I
LA_i+1	1.5	10/11	V/T	low sensitivity
LA_ii+1	1.5	9/11	T	low sensitivity
FYH_i+1	1	4/4		F are negative
FYH_ii+1	4	4/4		F are negative
R_i+1	1	4/4	G	
R_ii+1	1	3/4	R ^c^	low sensitivity
KR_i+1	6	10/11	G/S	low sensitivity
K_ii+1	6	4/7	G/R	low sensitivity, both positive and negative signals
P_i+1	2	2/2		
P_i-1	2	0/2		low sensitivity
P_i+1np	2	0/2		low sensitivity
P_i-1np	4	0/2		low sensitivity

^a^ The i+1 variants detect the successor to a certain amino acid type, whereas the ii+1 versions identify the selected amino acid itself and, very weakly, its successor. ^b^ Amino acids (i+1) which have proline as successor or are at the C-terminus (E18, I36, P37 and G76), and amino acids (ii+1) that have solvent or conformational exchanged amide peaks (M1, E24 and G53) were removed from the total number of that specific amino acid. ^c^ Side chains. ^d^ Only the appearance of the residue itself (i) is annotated in the ii+1 experiments because the signal strengths of the successors are usually low and might be difficult to detect in medium-sized proteins. Some of the MUSIC methods were very sensitive, like G_i+1, G_ii+1, A_i+1 and S_i+1, but these amino acid types are easily identified by standard 3D techniques as well. However, these MUSIC experiments can be useful for diminishing assignment ambiguities in overlap regions.

### 2.3. MUSIC Spectra of R17 and hNaa50p

Twenty four out of the 27 MUSIC variants that yielded good results on ubiquitin were acquired on the almost 20 kDa protein hNaa50p (Table 3). Three of the least sensitive experiments (A_ii+1, KR_i+1 and NQ_ii+1) were not conducted.

**Table 3:** Overview of the MUSIC experiments that were acquired on the two-medium sized proteins.

Variant	hNaa50p		R17				Comments
	Acquisition Time (h)	Signals	Acquisition Time (h)		Signals		
G_i+1 ^a^	4	8/10	n.d. ^b^		n.d.		side chain artefacts
G_ii+1 ^c^	3	4/9	n.d.		n.d.		
A_i+1 ^d^	9	7/7	12.5		12/13		
S_i+1	2	4/6	n.d.		n.d.		
S_ii+1	5	0/5	n.d.		n.d.		low sensitivity
N_i+1	4	4/11	4		5/6		
N_ii+1	8	1/11	n.d.		n.d.		low sensitivity
NQ_i+1	12	9/17	6		8/11		
DNG_i+1	6	17/30	5		16/21		G and some N are negative, some G are missing (R17)
DNG_ii+1 ^d^	10.5	8/28	17.5		17/20		E artefacts, G and some N are negative, D are strongest
EQG_i+1	5	8/22	5		20/24		N and D artefacts, also side chains, G are negative, Q are strongest
EQG_ii+1	10	9/23	8		14/24		N and D artefacts, also side chains, low G sensitivity (R17)
TA_i+1	3	0/14	n.d.		n.d.		low sensitivity
TA_ii+1	22	4/11	n.d.		n.d.		low sensitivity
VIA_i+1 ^d^	22	23/27	10		22/23		V has higher intensity than I
VIA_ii+1 ^d^	23.5	24/33	n.d.		n.d.		V has higher intensity than I
LA_i+1	1	12/20	n.d.		n.d.		low sensitivity
LA_ii+1	1	5/23	n.d.		n.d.		low sensitivity, even for A
FYH_i+1	10	6/18	8		7/8		all positive (R17), both positive and negative (hNaa50p)
FYH_ii+1 ^d^	19.5	7/15	16		5/7		all positive (R17), both positive and negative (hNaa50p)
W_i+1 ^d^	n.a. ^e^	n.a.	10		1/1		
W_ii+1 ^d^	n.a.	n.a.	15.5		1/1		
R_i+1	12	2/6	n.d.		n.d.		
KR_i+1	1	0/18	n.d.		n.d.		low sensitivity, test spectrum only
K_ii+1	1	0/11	n.d.		n.d.		low sensitivity, test spectrum only
P_i+1	20	2/5	n.a.		n.a.		

^a^ Amino acids (i+1) which have proline as successor or are at the C-terminus, and amino acids (ii+1) that have solvent or conformational exchanged amide peaks were removed from the total number of that specific amino acid. ^b^ Experiment not conducted ^c^ Only the appearance of the residue itself (i) is annotated in the ii+1 experiments since most of the successors are missing. ^d^ Shorter acquisition times might have been sufficient for these experiments. ^e^ Amino acid is missing from the protein sequence.

Several spectra showed small signal intensities, even at long acquisition times. The experiments which seem to be inappropriate for the use on this larger protein are: S_ii+1, N_ii+1 and those detecting threonine, arginine and lysine. Also, most of the ii+1 experiments, which are based upon 3D CBCANH, are significantly less sensitive than the i+1 version, which are based upon 3D CBCA(CO)NH, as expected from the sensitivity difference between these two 3D methods. However, the DNG, EQG and VIA ii+1 experiments resulted in rather good signal strengths.

Most of the artefacts and other features that were observed in the ubiquitin spectra were also present in the hNaa50p spectra. The only differences were that a few glutamate peaks appeared as artefacts in the DNG spectra, even though this was not expected since the DNG-sequence only includes one COSY step [15], and that the sign of the peaks in the FYH spectra could not be linked to the amino acid type.

Interestingly, the backbone assignment based on a repetition of the 3D standard experiments at high-field (800 MHz) were in agreement with the results obtained from CC(CO)NH and MUSIC at 600 MHz, and less than five additional peaks could be assigned using high-field NMR spectroscopy.

Only twelve of the MUSIC experiments, including those designed for tryptophan, were acquired on the 13 kDa protein R17 (Table 3), because of better signal resolution and strengths due to lower molecular mass making the assignment work less challenging than that of hNaa50p. Furthermore, the results from the MUSIC spectra on hNaa50p were taken into account so that the least sensitive methods were omitted.

**Figure 2:**
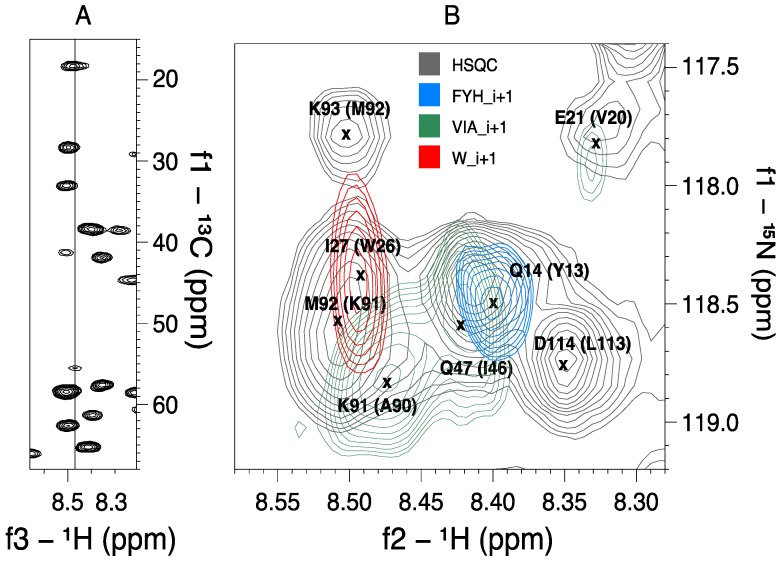
The significance of MUSIC experiments in an overlap region of a medium-sized protein. Panel (**A**) shows parts of the CBCA(CO)NH-plane of R17 at 118.5 ppm (^15^N). This is the region with the most shift redundancy in the ^15^N HSQC spectrum. (**B**) The superposition of HSQC and three MUSIC spectra illustrates how the latter can assist the identification of spin partners in the CBCA(CO)NH spectrum. For instance, the VIA_i+1 peak at approximately 8.47 ppm (^1^H) can easily be identified from the CBCA(CO)NH spectrum as an alanine successor (highlighted in **a**). The identity of the peaks and their predecessors are included in the HSQC spectrum.

Most of the selected MUSIC experiments worked very well on R17. Interestingly, the glycine signals were the least sensitive in the DNG and EQG experiments. Also, the peaks of the aspartate successors showed approximately the same signal strengths as the asparagine peaks in the DNG_ii+1 spectrum. That was surprising, because almost none of the successors were observable in any of the other ii+1 versions conducted on the medium-sized proteins. In the FYH experiments, all signals were positive. Thus, it was not possible to detect the amino acid identity directly from the spectrum. The presence of sign inversion in the spectra utilising L0S2-0 pulses (e.g., the FYH, DNG and EQG experiments) might indicate that the C^β^ and C*^γ^* pulses were not optimal. However, all protein spectra were conducted with identical acquisition parameters and, for instance, all peaks in the R17 FYH spectra were positive whereas they showed sign inversion in the two other proteins. An example of how MUSIC experiments helped to identify residues in an overlap region is shown in Figure 2, whereas Table 4 views the amino acids that could be assigned to the primary sequences of R17 and hNaa50p.

## 3. Experimental

### 3.1. Sample Preparation

99% ^13^C (5 mg) and 98% ^15^N labelled human ubiquitin dissolved in 50 mM potassium phosphate buffer (600 μL, containing 10% D_2_O), pH 5.8, was purchased from VLI Research Inc. (Malvern, PA, USA).

Uniformly ^13^C and ^15^N labelled R17 and hNaa50p were expressed and purified as previously described [21,26]. The concentration of both proteins was approximately 0.2 mM. R17 was dissolved in 20 mM sodium phosphate buffer (containing 10% D_2_O) pH 6.8, 0.1 M NaCl, 1 mM DTT and 0.05% NaN_3_ and hNaa50p in 50 mM sodium phosphate buffer (containing 10% D_2_O) pH 7.4, 0.1 M NaCl and 1 mM acetylcoenzyme A.

### 3.2. NMR Experiments on Ubiquitin

The spectra were collected at 298 K and 600.13 MHz (^1^H) on a Bruker Biospin AV600 spectrometer equipped with a superconducting actively shielded magnet. A 5 mm triple resonance (^1^H, ^13^C, ^15^N) inverse cryogenic probehead with z-gradient coils and cold ^1^H and ^13^C pre-amplifiers was used. The spectra were processed using Bruker Biospin’s TopSpin 1.3, and the resonance assignment was carried out in Cara [27].

The amide correlations were obtained from the ^15^N HSQC experiment [1,28]. Sequential backbone assignment was achieved from the spectra of the six standard 3D triple resonance experiments HNCA, HN(CO)CA, HNCO, HN(CA)CO, CBCA(CO)NH and HNCACB [29].

The 3D CC(CO)NH experiment [11,12] was acquired with isotropic mixing times of 22, 27 and 30 ms. The MUSIC experiments were collected using the pulses and parameters essentially as described in [13,14,15,16,17]; the pulse sequences and specific pulse shapes had been obtained from the Swiss Federal Institute of Technology, Zurich (ETHZ) [30].

**Table 4:** Overview of the amino acid sequences of hNaa50p (**a**) and R17 (**b**). Amino acids with assigned HN-peaks are highlighted. The columns denoted I and I-1 refer to the amino acid identity determined by the aliphatic chemical shifts and MUSIC experiments. Most unassigned residues are prone to exchange broadening. Note that the approach also works in stretches with repetitive amino acids (^21^EEEE^24^ and ^105^AAA^107^ in R17).

a									b					
Aa	I	I-1	Aa	I	I-1	Aa	I	I-1	Aa	I	I-1	Aa	I	I-1
M1			*A58*	A	G	*Q114*			M1			*F60*	FY	D
K2			*V59*	V	A	*I115*			*A2*	A		*T61*	T	FY
G3			C60			*S116*	ST	I	*R3*		A	*V62*		T
*S4*	ST	G	C61			*N117*		S	*G4*	G	R	*H63*		V
*R5*		S	*R62*			*E118*			*Q5*	EQ	G	*K64*		H
*I6*		R	*V63*	V	R	*S119*	S		*R6*		Q	*D65*	D	K
*E7*	E	I	*D64*		V	*A120*	A	S	*L7*		R	*R66*		D
*L8*		EQ	*H65*			*I121*	I	A	*E8*	EQ		*V67*	V	R
*G9*	G	L	*S66*	ST		*D122*	DN	I	E9			*N68*	N	V
*D10*		G	*Q67*		S	*F123*		DN	S10			*D69*		N
*V11*	V	DN	*N68*		Q	*Y124*			*L11*		S	*V70*	V	D
*T12*	T	V	*Q69*		N	*R125*			E12			*C71*		V
P13			*K70*		EQ	*K126*		R	*Y13*	FY	E	*A72*	A	
H14			*R71*			*F127*	FY	K	*Q14*		FY	*N73*		A
*N15*		H	*L72*		R	*G128*	G		*Q15*		Q	*G74*	G	N
*I16*		DN	*Y73*		L	*F129*		G	*F16*		Q	*E75*	EQ	G
*K17*		I	*I74*			*E130*			*V17*	V		*D76*	D	E
*Q18*	EQ	K	*M75*		I	*I131*		EQ	*A18*	A	V	*L77*	L	D
*L19*	L	EQ	*T76*	ST		*I132*		I	N19			*I78*	I	L
*K20*		L	*L77*		T	*E133*		I	*V20*	V	N	K79		
*R21*		K	*G78*	G		*T134*	T		*E21*	EQ	V	K80		
*L22*		R	*C79*		G	*K135*		T	*E22*		E	*N81*	N	K
*N23*		L	*L80*			*K136*		K	*E23*	EQ		N82		
*Q24*	EQ	DN	*A81*	A		*N137*		K	*E24*	EQ		H83		
*V25*	V	Q	P82			*Y138*		N	*A25*	A		H84		
*I26*	I	V	*Y83*		P	*Y139*		FY	*W26*	W	A	*V85*	V	
*F27*			*R84*			K140			*I27*		W	*E86*	EQ	
P28			*R85*			*R141*			*N28*	N	I	*N87*		E
*V29*	V		L86			*I142*	I	R	*E29*	EQ	N	*I88*		N
S30			*G87*	G	L	*E143*	EQ	I	*K30*		E	*T89*	T	I
*Y31*		S	*I88*		G	P144			*M31*		K	*A90*	A	T
N32			*G89*	G		*A145*	A	P	*T32*	T		*K91*		A
D33			*T90*	ST	G	*D146*		A	*L33*	L	T	*M92*		K
*K34*		DN	*K91*		T	*A147*	A	DN	*V34*	V	L	*K93*		
*F35*		K	*M92*		K	*H148*	H	A	*A35*	A	V	*G94*	G	K
Y36			*L93*			*V149*	V		*S36*	S	A	*L95*		G
K37			*N94*		L	*L150*		V	*E37*	EQ	S	*K96*		L
*D38*			*H95*	H		Q151			*D38*	D	E	*G97*	G	K
*V39*	V		*V96*	V	H	*K152*			*Y39*	FY	D	*K98*		G
*L40*		V	*L97*		V	*N153*			*G40*	G	FY	*V99*	V	K
*E41*	EQ		*N98*		L	*L154*			*D41*	DN	G	*S100*	S	V
*V42*	V	EQ	*I99*			*K155*		L	*T42*	T	D	*D101*	D	S
*G43*		V	*C100*		I	*V156*	V	K	*L43*		T	*L102*		D
*E44*		G	*E101*	EQ		P157			*A44*	A	L	*E103*	EQ	L
*L45*		EQ	*K102*		EQ	*S158*	ST	P	*A45*	A	A	*K104*		E
*A46*	A	L	*D103*		K	*G159*	G	S	*I46*		A	*A105*	A	K
*K47*		A	*G104*	G	DN	*Q160*		G	*Q47*	EQ	I	*A106*	A	A
*L48*			*T105*	ST	G	*N161*	DN	EQ	*G48*	G	Q	*A107*	A	A
*A49*	A		*F106*		T	*A162*	A	N	*L49*		G	*Q108*	EQ	A
*Y50*		A	*D107*	DN		D163			*L50*		L	*R109*		Q
*F51*			*N108*			V164			*K51*			*K110*		R
*N52*			*I109*			Q165			*K52*		K	*A111*	A	
*D53*			*Y110*			K166			*H53*	H	K	*K112*		A
*I54*			*L111*			T167			*E54*	EQ	H	*L113*		K
*A55*	A	I	H112			D168			*A55*	A	E	*D114*	D	L
*V56*	V	A	*V113*			N169			*F56*		A	*E115*	EQ	D
*G57*	G	V							*E57*	EQ	FY	*N116*	DN	E
									*T58*	T	E	*S117*	S	N
									*D59*	D	T	*A118*	A	S

### 3.3. NMR Experiments on R17 and hNaa50p

^15^N HSQC, standard 3D experiments and CC(CO)NH with an isotropic mixing time of 28 ms were acquired on the two medium-sized proteins at 600 MHz. Selections of twelve and 24 MUSIC experiments were conducted on R17 and hNaa50p, respectively. The spectra were collected at 298 K for R17 and at 310 K for hNaa50p, processed with TopSpin 1.3 and analyzed in Cara [27]. The chemical shifts were directly (^1^H) and indirectly (^13^C, ^15^N) referenced to DSS. For result comparison, the six standard 3D experiments for hNaa50p were repeated on a Varian Inova 800 NMR spectrometer with a 5-mm triple resonance probe (Swedish NMR centre, University of Gothenburg), processed with NMRPipe [31] and analyzed in Cara [27].

## 4. Conclusions

In a situation where high-field NMR spectroscopy was not available, the preparation of several samples was not practical and the standard methods were not sufficient, the following approach showed to be useful for the backbone assignment of two medium-sized proteins. First, the acquisition of a 3D CC(CO)NH spectrum using a long isotropic mixing time (27–30 ms), which allows for the detection of the remote aliphatic carbon shifts resulting in the identification of up to ten amino acid types. Second, the use of MUSIC variants that are most sensitive and that make it possible to identify those amino acid types that due to few aliphatic carbons are hard to detect by CC(CO)NH. These MUSIC experiments are the ones designed for the selection of asparagine, aspartate, glutamine, glutamate, valine, isoleucine, phenylalanine, tyrosine, histidin and probably tryptophan, especially the i+1 versions which are the most sensitive ones. This combination of experiments allows for the amino acid type identification of a large majority of peaks in the ^15^N HSQC spectrum, so that the 3D standard experiments can be used for the linkage of the right sequential pairs. Even though these methods cannot identify all peaks in a spectrum, especially in the most overlapping regions, this approach reduces the number of ambiguous peak identities and helps simplifying the assignment work. The extra time requirement for these methods is approximately 4–10 instrument days, depending on the size of the protein. However, the purification of several samples and the re-run of spectra at higher magnetic field strength or of partial deuterated samples could easily be in the same time range.

Although our approach can be used only for molecular weights up to 15–25 kDa and most kinases are larger, the strategy may be useful in the studies of kinases and their interaction partners. For instance, small kinases and their interaction with signal-initiating receptor ligands or targets may be examined directly; whereas domains of some of the larger kinases may be studied independently of the full-length proteins.
